# Ego-resiliency and perceived aggressive behavior in Chinese college students: the roles of employment anxiety and gender

**DOI:** 10.3389/fpsyg.2026.1933081

**Published:** 2026-09-08

**Authors:** Caixiang Chen

**Affiliations:** 1 College of Education, Zhejiang Normal University, Jinhua, China; 2 Student Affairs Office, Zhejiang Wanli University, Ningbo, China

**Keywords:** College student, ego-resiliency, employment anxiety, gender, perceived aggressive behavior

## Abstract

Given the rising incidence of campus violence and conflicts, aggressive behavior among college students has drawn considerable scholarly attention. Few studies, however, has examined the mediating and moderating mechanisms underlying the relationship between ego-resiliency and aggressive behavior. The current study examined the mediating effect of employment anxiety and the moderating effect of gender. A total of 925 Chinese college students (384 males; M_*age*_ = 19.48) completed questionnaires regarding ego-resiliency, employment anxiety, and aggressive behavior. Results showed that ego-resiliency was significantly and negatively associated with perceived aggressive behavior. This relationship was a partially mediated by employment anxiety. Furthermore, the indirect effect was moderated by gender. The simple slope tests showed that the relationship between ego-resiliency and employment anxiety was stronger for females, relative to the males. These findings highlight gender differences and support the importance, as noted in the existing literature, of incorporating ego-resiliency and employment anxiety into interventions and prevention efforts targeting perceived aggressive behavior.

## Introduction

Given the rising frequency of campus violence and conflicts, aggressive behavior among college students has garnered considerable scholarly attention (e.g., Wang et al., 2023; Xu et al., 2024). Aggressive behavior is defined as any act directed toward another individual with the immediate intent to cause harm (Anderson and Bushman, 2002). Studies have shown that such behavior can have severe repercussions for both perpetrators and victims, including substance abuse, depression, and suicide (Blain-Arcaro and Vaillancourt, 2017; Gvion and Apter, 2011; Zhou et al., 2022). Therefore, it is critical to investigate the factors and mechanisms underlying aggressive behavior among college students, so as to provide empirical evidence for developing effective intervention strategies.

However, prior research in China has predominantly focused on environmental correlates, such as negative life events and peer rejection (Xie et al., 2026; Yuan and Wang, 2026), whereas studies exploring the link between ego functions—particularly ego-resiliency—and aggressive behavior remain scarce. Although research has found that ego-resiliency negatively predicted aggressive behavior (e.g., Lee et al., 2022), the mediating processes that might explain this association, along with possible moderators that could strengthen or weaken it, are still largely unknown, especially within the Chinese cultural context. Therefore, based on ego-resiliency theory, this study would examine the mediating and moderating mechanisms between ego-resiliency and aggressive behavior among Chinese college students.

### Ego-resiliency and aggressive behavior in the Chinese context

Ego-resiliency refers to an individual’s dynamic ability to adaptively modulate ego-control in response to environmental demands, thereby maintaining or restoring systemic equilibrium (Block and Kremen, 1996). This trait exists on a continuum, ranging from high to low resiliency. According to ego-resiliency theory (e.g., Block and Block, 1980, 2006), highly resilient individuals are able to cope resourcefully with changing circumstances and unexpected events, accurately appraise the fit between situational requirements and available behavioral options, and flexibly deploy existing problem-solving strategies. In contrast, individuals low in ego-resiliency exhibit limited adaptability, fail to adjust to shifting situational demands, tend to become rigid or disorganized under stress, and struggle to recover from adverse experiences. Previous research has found that ego-resiliency negatively predicted aggressive behavior (e.g., Lee et al., 2022), likely because those with higher ego-resiliency are better able to flexibly regulate their ego-control to match contextual needs, thereby reducing the likelihood of aggressive responses.

Nevertheless, while Western research—grounded in individualistic value orientations—has consistently established the predictive role of ego-resiliency in aggressive behavior (e.g., Lee et al., 2022; for a review, see Bohane et al., 2017), this topic remains underexplored in China. Most domestic studies have instead concentrated on environmental predictors, such as negative life events and peer rejection, in relation to aggressive behavior among Chinese college students (Xie et al., 2026; Yuan and Wang, 2026). Moreover, Chinese society is deeply shaped by Confucian culture, which emphasizes collective-oriented values and strong moral standards; consequently, aggressive acts are likely to attract pronounced social disapproval (Chen, 2023; Chen and French, 2008). Indeed, individuals in collectivist cultural contexts tend to exhibit lower levels of aggression than their counterparts in individualistic cultures (Chen, 2023; Lansford et al., 2018). More importantly, according to the model of culture and personality (Church, 2000; Triandis and Suh, 2002), personality traits exist in all cultures, but in collectivist cultures, they have less influence on behavior than in individualist cultures. Therefore, compared to Western culture, the relationship between ego-resiliency and aggressive behavior may be weaker in the Chinese cultural context. In light of this, it is necessary to explore the relationship between ego-resiliency and aggressive behavior within the Chinese cultural context.

### Employment anxiety as a mediator

Employment anxiety is defined as a psychosomatic reaction to employment- or job-seeking-related pressures, encompassing negative emotional states such as tension, restlessness, worry, and fear, as well as physiological and behavioral responses including sleep disturbances, increased heart rate, chest tightness, and concentration difficulties (Chen and Zeng, 2021). Previous researchers have not examined the relationship between ego-resiliency and employment anxiety, but studies have found that ego-resiliency is negatively associated with anxiety (Badura-Brzoza et al., 2025; Skalski et al., 2021). According to ego-resiliency theory (Block and Block, 1980), individuals with low ego-resiliency lack the flexibility to modulate their ego-control appropriately, rendering them more prone to anxiety when confronted with highly competitive environments. As Block and Block (2006) further noted, relatively unresilient or vulnerable individuals exhibit little adaptive flexibility and tend to feel anxious under competing demands. In recent years, China has witnessed a sustained increase in the number of university graduates, resulting in an uncertain and highly competitive labor market (e.g., Jin et al., 2024). In this context, those with low ego-resiliency are likely to experience greater employment anxiety than their more resilient counterparts.

Furthermore, employment anxiety may serve as a significant positive predictor of aggressive behavior. According to the frustration–aggression model (e.g., Berkowitz, 1989; Polman et al., 2007), aggression arises from frustration—when internal or external obstacles block goal attainment, negative emotions are elicited. These heightened negative emotions, such as anxiety and anger, in turn increase the likelihood of aggressive responses, whether as defensive reactions or as attempts to harm the source of frustration. The greater the importance of the thwarted goal, the stronger the frustration, and consequently, the stronger the impulse to aggress (Dollard et al., 1939). Put differently, when individuals are unable to avoid or withdraw from a given situation, anxiety may precipitate aggressive behavior (Bubier and Drabick, 2009). Previous studies have found that anxiety is positively related to aggressive behavior (e.g., Jiang et al., 2026; Zhang et al., 2022). Therefore, drawing on both theoretical frameworks and prior empirical findings, we hypothesized that employment anxiety would be significantly and positively related to aggressive behavior.

Taken together, based on the theoretical and empirical evidence, we hypothesized that employment anxiety would mediate the relationship between ego-resiliency and aggressive behavior.

### Gender as a moderator

Although the existing literature has demonstrated that ego-resiliency is negatively correlated with employment anxiety and aggressive behavior (e.g., Block and Block, 2006; Lee et al., 2022), not all individuals exhibit high levels of these outcomes. Some studies have examined gender differences in ego-resiliency (Block and Block, 2006; Taylor and Spinrad, 2018). For instance, in a 30-year longitudinal study, Block and Block (2006) found that individual differences in ego-resiliency can be identified as early as childhood and persist over the subsequent two decades. However, for girls, childhood ego-resiliency—or conversely, vulnerability—did not predict their ego-resiliency during adolescence or adulthood, with this instability becoming particularly pronounced after they entered adolescence. In contrast, a 13-year longitudinal study found that boys exhibited lower levels of ego-resiliency and experienced greater declines during adolescence, whereas girls demonstrated higher ego-resiliency (Chuang et al., 2006). In addition, studies have found that females tend to experience higher levels of employment anxiety than males (Wang and Hu, 2025; Yang et al., 2025), possibly because females are more sensitive to age-related employment pressures. Given the inconsistent findings concerning gender differences in ego-resiliency, we intend to explore the moderating role of gender in the relationship between ego-resiliency and employment anxiety on an exploratory basis, without formulating any *a priori* hypotheses.

Furthermore, given the well-established gender differences in aggressive behavior, males tend to exhibit higher levels than females (e.g., Björkqvist, 2018; Padgett and Tremblay, 2020). This pattern may be explained by differing social representations: women typically hold an “expressive” view, perceiving aggression as a loss of self-control, whereas men hold an “instrumental” view, regarding aggression as a means of controlling others (Campbell and Muncer, 1994). In addition, considering the documented gender differences in ego-resiliency and the mixed findings in prior research, we also explored whether gender moderates the relationship between ego-resiliency and aggressive behavior, again on an exploratory basis without formulating specific hypotheses. Moreover, in light of previous findings that employment anxiety and aggressive behavior each show gender differences (e.g., Björkqvist, 2018; Wang and Hu, 2025), we explored the potential moderating role of gender in their association on an exploratory basis.

### The present study

As mentioned above, previous research has two major limitations. First, few studies have simultaneously examined the mediating and moderating mechanisms in the relationship between ego-resiliency and aggressive behavior. Second, most past studies exploring the relationship between ego-resiliency and aggressive behavior have been conducted within Western cultural contexts. Importantly, findings from studies conducted in Western cultural contexts may not necessarily apply to Chinese culture. For example, because Chinese society emphasizes group orientation and adherence to moral discipline, individuals in China exhibit lower levels of aggression than their counterparts in individualistic cultures (Chen, 2023).

**FIGURE 1:**
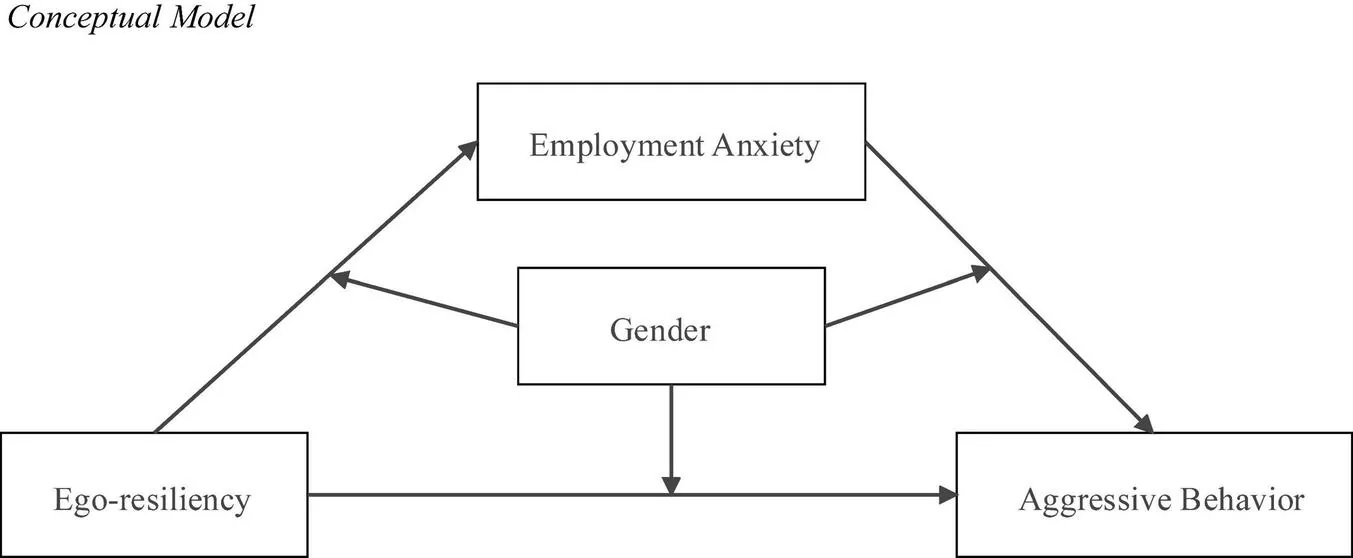
Conceptual model.

Therefore, in this study, we aim to explore the mediating mechanisms in the relationship between ego-resiliency and aggressive behavior among Chinese college students. Based on the above literature review, we proposed three hypotheses (see Figure 1): (1) ego-resiliency would be significantly negatively correlated with aggressive behavior; (2) employment anxiety may serve as a mediating variable in the relationship between ego-resiliency and aggressive behavior; and (3) gender would play a significant moderation role between ego-resiliency, employment anxiety and aggressive behavior.

## Materials and methods

### Participants

The present study used convenience sampling to recruit students from two general universities in Zhejiang Province, China. One university specializes in science and engineering, while the other is a comprehensive university. Both universities are classified as mid-tier institutions within Zhejiang Province. As the number of college graduates continues to rise each year, students in every grade may experience employment anxiety. With this in mind, a survey was conducted among students in all four grades. Although we anticipated data from 1,000 participants, some students were absent, and some questionnaires were discarded because they were completed carelessly (e.g., with straight-line responses). Finally, 925 college students (384 males) completed the survey, with ages ranging from 17 to 24 (M_*age*_ = 19.48, SD = 1.27). Of these, 389 were freshmen (146 males), 266 were sophomores (116 males), 197 were juniors (72 males) and 73 were seniors (50 males). Almost all participants are Han ethnic nationality, which is primary ethnic nationality (over 95% of the population) in China. In addition, a statistical power analysis conducted using G*Power indicates that the required sample size to detect effect sizes ranging from small to large (i.e., 0.02–0.35; Funder and Ozer, 2019) should be between 31 and 485. Therefore, the sample size in this study provides sufficient statistical power. Data were collected in early May 2025.

### Measures

#### Ego-resiliency

The Ego-resiliency Scale (ER89; Block and Kremen, 1996) is a 14-item questionnaire (e.g., “I get over my anger at someone reasonably quickly”). The Chinese version revised by Chen et al. (2020). Each item is rated on a 4-point scale (1 = disagree very strongly, 4 = agree very strongly). We calculated the mean scores with high scores indicating high levels of ego-resiliency. This measure has been shown to be reliable and valid with Chinese students (e.g., Sun et al., 2026). Cronbach’s α was 0.90.

#### Employment anxiety

The Chinese version of Employment Anxiety Scale (Wan et al., 2024) is a 7-item questionnaire (e.g., “When I think about getting employment after graduation last week, my heart beats fast and I feel very nervous”). Each item is rated on a 7-point scale (1 = strongly disagree, 7 = strongly agree). We calculated the mean scores with high scores indicating high levels of employment anxiety. This measure has been shown to be reliable and valid (e.g., Obi-Anike et al., 2026). Cronbach’s α was 0.91.

#### Aggressive behavior

The Brief Aggression Questionnaire (BAQ; Webster et al., 2014) is a 12-item questionnaire (e.g., “There are people who pushed me so far that we came to blows”). The Chinese version revised by Shi et al. (2025). Each item is rated on a 5-point scale (1 = extremely uncharacteristic of me, 5 = extremely characteristic of me). We calculated the mean scores with high scores indicating high levels of perceived aggressive behavior. This measure has been shown to be reliable and valid with Chinese students (e.g., Shi et al., 2025). Cronbach’s α were 0.81.

#### Procedure

The questionnaire was administered by members of our trained team. This study has been approved by the Ethics Review Committee of the Zhejiang Wanli University. All classroom participants completed paper questionnaires. The classes to be tested were all contacted prior to the test. Before collecting data, verbal informed consent was obtained from each participant and their guardians (i.e., student counselors). We informed participants that they could withdraw from the study at any time. It took approximately 20 min to complete the questionnaire. To ensure that the data collection process met psychometric standards, efforts were focused primarily on the following areas: First, a standardized administration procedure was adopted, with trained interviewers administering the same paper-and-pencil questionnaire in a designated classroom at a fixed time. Second, the items on the questionnaire were randomly ordered to prevent participants from guessing the questions. Finally, any questionnaire with identical answers for all items was deemed invalid. In addition, when collecting the questionnaires, the experimenter would review them; if any items were missing, they would remind the participants to complete them.

### Data analysis

We first calculated the correlations and descriptive statistics by using SPSS (version 26.0). Then, the moderated mediation model was analyzed by PROCESS macro (version 4.1; Hayes, 2022). The 95% confidence intervals (CI) of bias-corrected bootstrap whose bootstrap samples was 5,000.

## Results

### Common method variance

Using the Harman single factor test method, the results showed that there were 6 factors with characteristic roots greater than 1. The first factor explains 23.14% of the total variation, which is far less than the critical value of 40%, so there was likely no common method deviation (Zhou and Long, 2004).

### Descriptive statistics and correlation analyses

The descriptive statistics and correlations are presented in Table 1. As hypothesized, ego-resiliency was negatively correlated with employment anxiety and perceived aggressive behavior. Employment anxiety was positively correlated with perceived aggressive behavior. Gender was significantly associated with employment anxiety and perceived aggressive behavior. Age was not significantly correlated with employment anxiety, and perceived aggressive behavior.

**TABLE 1:** Descriptive statistics and correlations among main study variables.

Variables	1	2	3	4	5	M	SD
1. Gender	–					–	–
2. Age	0.09**	–				19.48	1.27
3. Grade	0.10**	0.82***	–			1.95	0.97
4. Ego-resiliency	0.06^†^	−0.04	−0.07*	–		2.95	0.48
5. Employment anxiety	−0.07*	0.06^†^	0.06^†^	−0.20***	–	3.37	1.27
6. Perceiver aggressive behavior	0.18***	−0.02	0.00	−0.14***	0.35***	2.52	0.60

Gender coded as female = 0, male = 1.

† *p* < 0.1, *p < 0.05, **p < 0.01, ***p < 0.001.

### Testing the mediating role of employment anxiety

First, we standardized all variables, except for gender. We tested the mediating model by using PROCESS macro (model 4). The results in Table 2 showed that ego-resiliency was negatively associated with perceived aggressive behavior (β = −0.08, SE = 0.03, *p* = 0.006), after controlling for age, gender, and grade. After controlling for age, gender, and grade, ego-resiliency was negatively associated with employment anxiety (β = −0.19, SE = 0.03, *p* < 0.001), and employment anxiety was positively associated with perceived aggressive behavior (β = 0.35, SE = 0.03, *p* < 0.001). The mediating effect of ego-resiliency on perceived aggressive behavior through employment anxiety was significant (standardized indirect effect = −0.07, SE = 0.02, 95% CI [−0.10, −0.04]). The mediation effect accounted for 44.33% of the total effect.

**TABLE 2:** Mediated role of employment anxiety on relation between ego-resiliency and perceiver aggressive behavior.

Predictor	β	SE	95% CI for β	*t*-value
Employment anxiety				
Gender	−0.13	0.07	[−0.26, −0.01]	−2.04*
Age	0.02	0.06	[−0.09, 0.13]	0.41
Grade	0.03	0.06	[−0.08, 0.13]	0.61
Ego-resiliency	−0.19	0.03	[−0.26, −0.13]	−5.97***
*R* ^2^	0.05			
*F*	11.29***			
Perceiver aggressive behavior				
Gender	0.44	0.06	[0.32, 0.56]	7.12***
Age	−0.08	0.05	[−0.18, 0.03]	−1.45
Grade	0.02	0.05	[−0.08, 0.12]	0.40
Employment anxiety	0.35	0.03	[0.29, 0.41]	11.31***
Ego-resiliency	−0.08	0.03	[−0.15, −0.02]	−2.76**
*R* ^2^	0.18			
*F*	38.44***			

CI, confidence interval. *p < 0.05, **p < 0.01, ***p < 0.001.

### Testing the moderating role of gender

**FIGURE 2:**
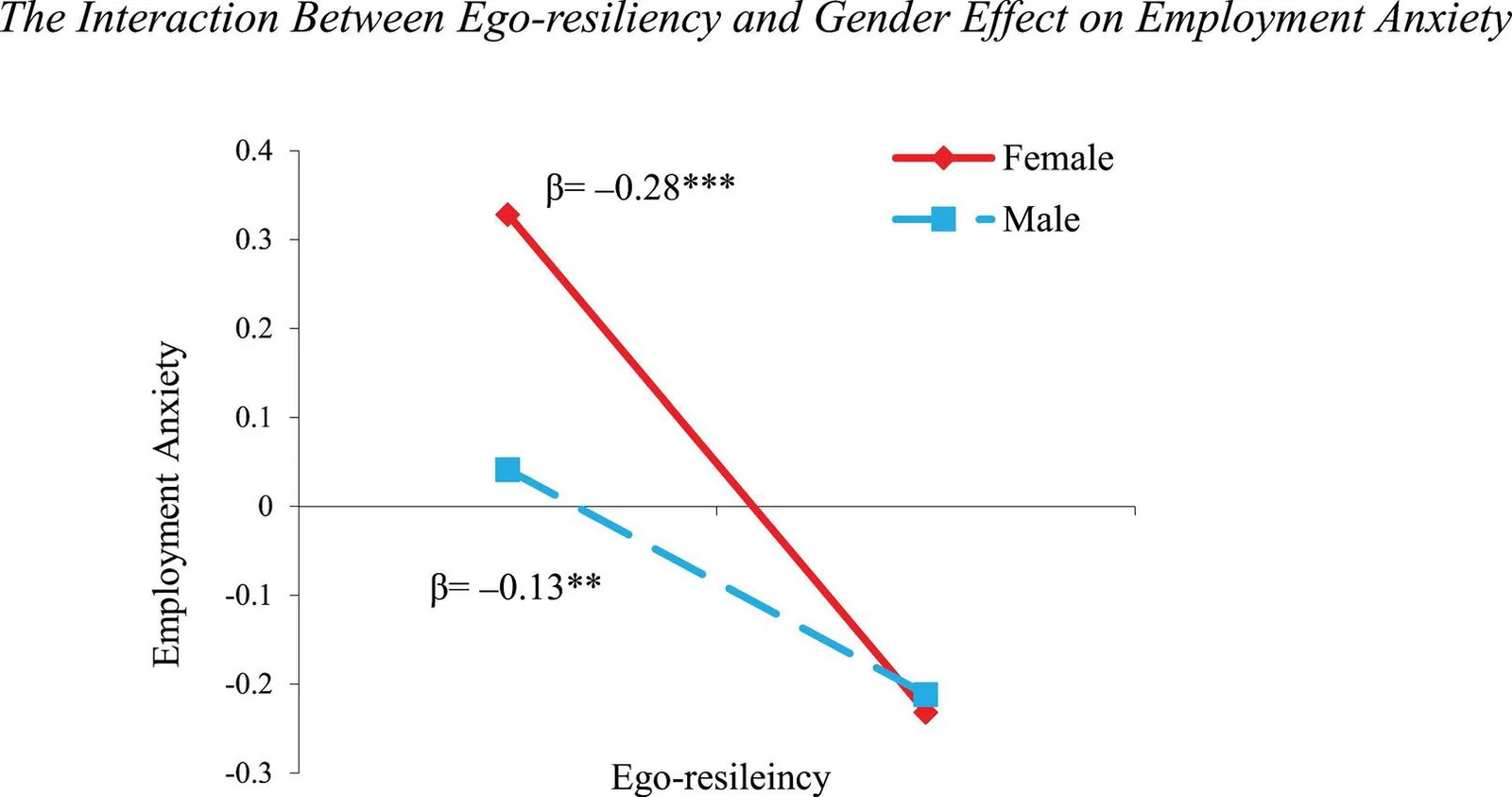
The interaction between ego-resiliency and gender effect on employment anxiety. **p < 0.01, ***p < 0.001.

Then, we tested the moderated mediation model by using PROCESS macro (model 59). As shown in Table 3, the interaction between ego-resiliency and gender had a significant moderating effect on employment anxiety (β = 0.15, SE = 0.06, *p* = 0.02). Further simple slope analysis (see Figure 2) showed that the relationship between ego-resiliency and employment anxiety was stronger for females (β = −0.28, SE = 0.05, *p* < 0.001), relative to the males (β = −0.13, SE = 0.04, *p* = 0.003). For females, ego-resiliency had a significantly and negatively predictive effect on perceived aggressive behavior through employment anxiety, standardized indirect effect = −0.08, SE = 0.02, 95% CI [−0.12, −0.04]. This indirect effect was significant for males, standardized indirect effect = −0.05, SE = 0.03, 95% CI [−0.10, −0.06]. In addition, the interaction between ego-resiliency and gender had a nonsignificant moderating effect on perceived aggressive behavior (β = 0.03, SE = 0.06, *p* = 0.66). The interaction between employment anxiety and gender had a nonsignificant moderating effect on perceived aggressive behavior (β = 0.11, SE = 0.06, *p* = 0.07).

**TABLE 3:** Testing the moderated mediation effect of ego-resiliency on perceiver aggressive behavior.

Predictor	β	SE	95% CI for β	*t*-value
Employment anxiety				
Gender	−0.13	0.07	[−0.26, −0.00]	−2.03*
Age	0.02	0.06	[−0.09, 0.13]	0.39
Grade	0.03	0.06	[−0.08, 0.14]	0.57
Ego-resiliency	−0.28	0.05	[−0.38, −0.18]	−5.73***
Ego-resiliency × gender	0.15	0.06	[0.03, 0.28]	2.37*
*R* ^2^	0.05			
*F*	10.20***			
Perceiver aggressive behavior				
Gender	0.44	0.06	[0.32, 0.56]	7.12***
Age	−0.08	0.05	[−0.18, 0.03]	−1.47
Grade	0.02	0.05	[−0.08, 0.12]	0.41
Employment anxiety	0.29	0.04	[0.21, 0.38]	6.76***
Ego-resiliency	−0.12	0.05	[−0.21, −0.02]	−2.45*
Ego-resiliency × gender	0.05	0.06	[−0.07, 0.17]	0.79
Employment anxiety × gender	0.11	0.06	[−0.01, 0.24]	1.83^†^
*R* ^2^	0.18			
*F*	28.01***			

CI, confidence interval.

† *p* < 0.1, *p < 0.05, ***p < 0.001.

### Sensitivity analysis

To test whether the total effect of ego-resiliency on perceived aggressive behavior differs by gender, we tested the moderated model by using PROCESS macro (model 1). The results showed that after controlling for age and grade, the interaction between ego-resiliency and gender had a nonsignificant moderating effect on perceived aggressive behavior (β = 0.08, SE = 0.06, *p* = 0.22).

## Discussion

The present study specifically examined whether employment anxiety would mediate the relationship between ego-resiliency and perceived aggressive behavior, and whether the relationship between ego-resiliency and employment anxiety, as well as between ego-resiliency and perceived aggressive behavior, would be moderated by gender. The results indicated that employment anxiety partially mediated the relationship between ego-resiliency and perceived aggressive behavior, and that the interaction between ego-resiliency and gender on employment anxiety was significant.

### Ego-resiliency and perceived aggressive behavior

The present study found a significant negative correlation between ego-resiliency and perceived aggressive behavior. This finding supported Hypothesis 1, which is consistent with previous studies (Lee et al., 2022; for a review, see Block and Block, 1980). This association can be understood through the adaptive function of ego-resiliency as a personality resource that enables individuals to modulate the level and habitual expression of ego-control, thereby facilitating optimal engagement with and adaptation to their immediate and long-term environments (Klohnen, 1996). Conversely, individuals low in ego-resiliency lack such flexibility, becoming locked into rigid response patterns—such as undercontrol—which, under environmental stress, may manifest as aggressive behavior. In contrast, those with high ego-resiliency are less prone to peer conflicts and tend to employ positive coping strategies when conflicts do occur (Xie et al., 2016). Collectively, the results suggested that ego-resiliency may be a predictor of perceived aggressive behavior.

### The mediating role of employment anxiety

This study further revealed that employment anxiety mediated the relationship between ego-resiliency and perceived aggressive behavior. This finding supported Hypothesis 2. According to ego-resiliency theory (e.g., Block, 2002; Block and Block, 1980), resilient individuals tend to remain energetic when confronting novel or stressful situations and generate more alternative solutions when faced with obstacles. Specifically, when encountering employment pressure, individuals with high ego-resiliency are better able to proactively plan their careers, set clear employment goals, and persistently strive toward them. In contrast, those with low ego-resiliency are more prone to employment anxiety, as they lack effective strategies—such as goal-setting—and the sustained effort needed to achieve their objectives.

Complementarily, the frustration–aggression model (e.g., Berkowitz, 1989; Polman et al., 2007) posits that frustrations are aversive events that elicit aggressive tendencies only when they generate negative emotions. That is, thwarting experiences trigger negative affective states—which individuals typically seek to alleviate or eliminate—and it is precisely these negative feelings that give rise to aggressive inclinations (Berkowitz, 1989). In the context of intense job competition, individuals are likely to encounter setbacks in their job search, which in turn provoke employment anxiety; it is these negative emotional responses that ultimately contribute to perceived aggressive behavior.

In summary, the present findings suggested that employment anxiety may represent the key mechanism accounting for the relationship between ego-resiliency and perceived aggressive behavior. Theoretically, this finding enriches our understanding of how ego-resiliency relates to aggressive behavior.

### The moderating role of gender

The present study further revealed that gender moderated the relationship between ego-resiliency and employment anxiety, partially supporting Hypothesis 3. Specifically, females reported higher levels of employment anxiety than males (Wang and Hu, 2025; Yang et al., 2025), a difference that may be attributable to females’ greater sensitivity to employment-related issues and the prevalence of gender bias in the job market (Wang and Hu, 2025). Additionally, females may experience greater anxiety regarding childbearing compared to their male counterparts. As a personality resource that serves the ego, ego-resiliency enables individuals to employ flexible problem-solving strategies and adapt more effectively to environmental demands (Block, 2002; Block and Block, 1980). Consequently, the negative association between ego-resiliency and employment anxiety is more pronounced among females than among males. Moreover, given gender’s moderating role in the relationships among ego-resiliency, employment anxiety, and aggressive behavior, the indirect pathway from ego-resiliency to aggressive behavior through employment anxiety also varies by gender—specifically, this mediating effect was weaker for males than for females. This may be explained by the cumulative, powerful, and pervasive effects of differential socialization experiences traditionally afforded to females, which shape adaptive strategies particularly when individuals encounter fundamental life transitions (Block and Block, 2006). Therefore, the buffering effect of ego-resiliency against employment anxiety appears stronger in females than in males.

In addition, this study found that gender did not moderate the relationship between ego-resiliency and perceived aggressive behavior, nor the relationship between employment anxiety and perceived aggressive behavior. This may be attributable to the collectivist orientation prevalent in Chinese society, which not only encourages adherence to external norms but also demands internalization of societal expectations regarding appropriate conduct (Chen and French, 2008). In such a cultural context, aggressive, disruptive, or defiant behaviors are strictly proscribed, as they are perceived as threats to social harmony and may invite severe social censure (Chen, 2023; Chen and French, 2008). Therefore, both males and females are motivated to conform to social norms and restrain any inclination toward perceived aggressive behavior, thereby attenuating potential gender differences in this regard.

### Limitations and future directions

Several limitations of this study should be acknowledged. First, the cross-sectional design precludes causal inferences regarding the relationships among the variables. Future longitudinal or experimental studies are therefore warranted to corroborate the mediating role of employment anxiety in the link between ego-resiliency and perceived aggressive behavior. Moreover, this study did not control for potential confounding variables, such as academic major (e.g., humanities vs. sciences) or family socioeconomic status. Accordingly, the present findings should be interpreted with caution, and future research should incorporate these variables as covariates to validate our results. Second, all measurements were based on self-reports, which affects the validity of this study. Since it is difficult to avoid common-method bias in self-reported data, therefore future research should employ multiple data sources (e.g., peer rating and behavioral measurements). Finally, the sample was drawn exclusively from college students at two universities in China, which limits the generalizability of our conclusions. Future research should examine whether these relationships hold among students from other regions of China (e.g., the western areas) and, more broadly, across different cultural contexts.

### Implications

This study offers several practical implications for college students and educators. Theoretically, our findings underscore the importance of ego-resiliency as a key personality construct and contribute to a deeper understanding of the mechanisms linking ego-resiliency to perceived aggressive behavior. Specifically, employment anxiety emerged as a mediating pathway through which ego-resiliency relates to aggressive behavior. From a practical standpoint, interventions targeting employment anxiety may help reduce aggressive tendencies among individuals with high anxiety levels. For instance, career planning services—such as goal identification, feasible plan development, and skill acquisition—could alleviate employment anxiety and subsequently diminish perceived aggressive behavior.

Additionally, gender moderated the relationship between ego-resiliency and employment anxiety, with this association being stronger for females than for males. This suggests that particular attention should be directed toward female students with elevated employment anxiety. Tailored career development counseling that assists them in identifying suitable professional trajectories may be especially beneficial.

It is important to note, however, that these practical recommendations rest on the assumption of causal relationships among the variables. Given that correlation does not imply causation, the present findings should be interpreted with caution, and the proposed implications should be considered preliminary pending further causal evidence.

## Conclusion

The findings revealed that employment anxiety partially mediated the relationships between ego-resiliency and perceived aggressive behavior among Chinese college students. Furthermore, gender moderated the relationships between ego-resiliency and employment anxiety, with this relationship being more pronounced in females than in males. These findings underscore gender differences and highlight the importance—as previously noted in the literature—of integrating both ego-resiliency and employment anxiety into intervention and prevention efforts targeting perceived aggressive behavior. Additionally, the present study found that gender did not moderate the relationship between ego-resiliency and perceived aggressive behavior, nor the relationship between employment anxiety and perceived aggressive behavior.

## Data Availability

The raw data supporting the conclusions of this article will be made available by the authors, at reasonable request.
